# Application of Fuzzy AHP for Medication Decision Making in Iron-Chelating Medications for Thalassemia

**DOI:** 10.3390/pharmacy13030086

**Published:** 2025-06-15

**Authors:** Saeed Barzegari, Hosein Rostamian, Ehsan Firoozi-Majd, Ibrahim Arpaci

**Affiliations:** 1 Department of Paramedicine, Amol Faculty of Paramedical Sciences, Mazandaran University of Medical Sciences, Amol 48175-866, Iran; 2 Department of Medical Immunology, School of Medicine, Tehran University of Medical Sciences, Tehran 14167-53955, Iran; 3 Management Information Systems, College of Business Administration, Gulf University for Science and Technology, Mishref 7207, Kuwait; 4 Department of Software Engineering, Faculty of Engineering and Natural Sciences, Bandirma Onyedi Eylul University, Balikesir 10200, Turkiye; 5 Department of Computer Science and Engineering, College of Informatics, Korea University, Seoul 02841, Republic of Korea

**Keywords:** thalassemia, fuzzy AHP, medication decision making

## Abstract

Iron overload is a significant concern for patients with thalassemia and often necessitates the use of iron-chelating agents to mitigate the associated complications. Selecting the most appropriate chelation therapy from the available options is a complex decision for healthcare professionals. To support this decision-making process, this study investigates the application of the “Fuzzy Analytic Hierarchy Process” (FAHP) for medication selection in thalassemia patients requiring iron-chelation therapy. In this study, 20 hematologists participated, and matrices related to the FAHP model were used to evaluate three primary iron chelators: deferoxamine, deferasirox, and deferiprone. The results revealed that deferiprone was the most effective choice, while deferasirox outperformed the others in terms of cost and patient satisfaction. Notably, deferoxamine exhibits the highest rate of side effects, followed by deferiprone and deferasirox. The results obtained from the FAHP analysis indicated a consensus among experts and highlighted deferasirox as the optimal choice for treating chronic iron overload in thalassemia patients. The study demonstrates the practical applicability of the FAHP methodology in guiding informed decisions for iron-chelation therapy. It provides insights to help healthcare professionals optimize treatment strategies for patients with thalassemia.

## 1. Introduction

Thalassemia is a genetic disorder associated with defects in the hemoglobin chain [1]. Patients with beta thalassemia major and intermedia require lifelong blood transfusions, which can lead to iron overload and subsequent accumulation in organs such as the heart, liver, spleen, and pancreas [2,3]. Iron chelation therapy is critical to mitigate these complications, with early diagnosis and treatment being essential to prevent toxic effects [4].

Deferoxamine (DFO), deferiprone (DFP), and deferasirox (DFX) are iron chelator agents widely used in patients with iron overload [5]. DFO, introduced in 1960, is administered via subcutaneous infusion for 8–12 h weekly [2,6]. The first oral chelator introduced for clinical use was DFP [7]. DFX was introduced as the first-line treatment for patients older than 2 years with iron overload [8,9]. These three medications differ in several characteristics, including plasma half-life, cardiac iron load, liver iron concentration, serum ferritin levels, side effects, degree of patient satisfaction, and price [10].

Specialists must weigh multiple qualitative and quantitative factors when choosing an iron chelator. The FDA’s 2013 guidelines on structured benefit–risk assessment highlight the need for quantitative methods to integrate treatment trade-offs. It is suggested that the benefit–risk analysis be performed using multi-criteria decision analysis [11]. When the decision maker is faced with many options that are affected by multiple criteria, it is recommended to use the “Analytic Hierarchy Process” (AHP), which is one of the best multi-criteria decision-making methods [12]. AHP incorporates risks and benefits transparently by combining the importance of differences in the probability of results related to (treatment) alternatives [13,14]. This method is used in many fields of medicine and health sciences, and so far, it has been proven to be a competent method [15].

Since the decision-making process is explicit in the AHP, groups or individuals can comprehend and demonstrate the basis of their decisions using this method. However, unlike the AHP, the importance of different components of the decision is not transparent in the standard decision-making process [13]. In this method, the decision maker compares and weighs the criteria and sub-criteria that affect the priority of the alternatives. However, AHP is also criticized for not representing the imprecision and uncertainty of the style of human thought [16,17]. Fuzzy logic offers a more nuanced approach to addressing these biases, rather than relying on exact values. Fuzzy set theory was developed to address uncertainty resulting from vagueness and imprecision. Decision makers usually consider it more convenient to make interval judgments than decisions based on fixed values [18]. Chang implemented a new approach to handle the Fuzzy AHP (FAHP) by applying the triangular fuzzy numbers for the pairwise comparison scale of the FAHP [19].

Selecting the most appropriate iron chelator from available options—deferoxamine, deferiprone, and deferasirox—is a complex decision-making process for healthcare specialists. The differences in these agents’ characteristics, such as efficacy, side effects, patient satisfaction, and cost, require an informed selection process. Through FAHP, this research aims to bridge the existing gap in decision-making processes for thalassemia treatment and provide a systematic and informed approach for selecting iron-chelating medications tailored to the needs of thalassemia patients.

## 2. Materials and Methods

The application of spherical fuzzy sets in multi-criteria group decision making has attracted attention in various domains [20,21,22,23,24]. Although this methodology is increasingly being used, a significant gap remains in the current literature regarding the specific application of FAHP in medication decision making. This study was conducted in seven steps.

1. Review studies to determine the alternatives, criteria, and sub-criteria, and develop the AHP model.
2. Select experts according to the exclusion and inclusion criteria.
3. Determine the side effects of the alternatives and categorize them into three sub-criteria, namely mild, moderate, and severe, for the experts’ evaluation.
4. Train the experts to complete the pairwise comparison matrix.
5. Use the FAHP analysis to prioritize the alternatives, criteria, and sub-criteria [25].
6. Repeat step four in case of a consistency ratio higher than 0.1.
7. Conduct the agreement analysis of preferences between two groups of experts with either over or below 10 years of experience using the Bland–Altman test.

### 2.1. Study Group

The inclusion criteria for selecting experts in our study are as follows: the experts must be specialists in hematology and oncology with a minimum of three years of clinical experience in treating thalassemia patients with iron overload. According to the inclusion criteria, the group of experts consisted of 20 hematologists with at least three years of clinical experience and research on thalassemia in the hospital. The hematologists had experience prescribing these iron chelators to reduce the iron overload of thalassemia patients, and they were fully aware of the effects, side effects, and costs of these three drugs. Participants were informed of the AHP theory and the study’s objectives. Informed consent forms were obtained from each participant. The hematologists were divided into two groups: specialists with more than 10 years of experience and those with less than 10 years of experience.

### 2.2. Developing an AHP Model

**Figure 1:**
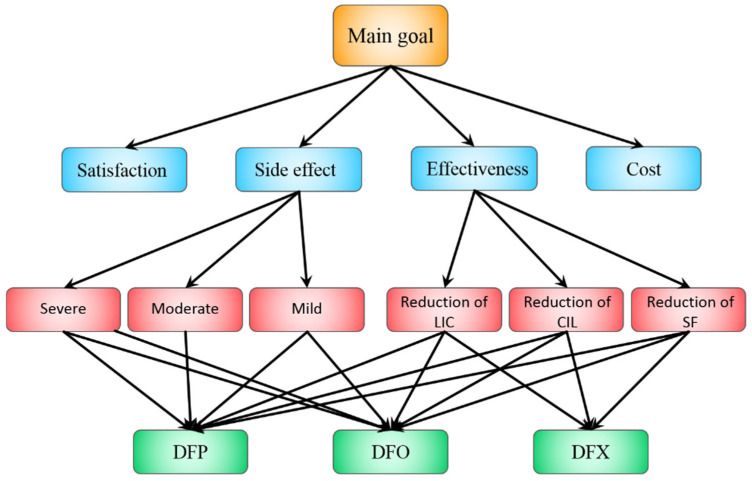
AHP model.

The first step in creating an AHP model is organizing our problem into a hierarchy. To find suitable criteria and sub-criteria for our study, we searched the PubMed and Scopus databases and then designed our AHP model based on these results. The hierarchical structure of the AHP model, as presented in Figure 1, is based on previous studies. Blue rectangles show criteria, red rectangles show sub-criteria, and green rectangles show alternatives.

Here, variables and decision criteria are divided into three primary levels:

Level one: Choosing the right chelator for thalassemia patients.

Level two: Criteria that are used for choosing the right chelator.

Level three: Sub-criteria that are used for selecting the drug.

The efficacy of drugs varies for different patients, and a few molecular or clinical factors can help us predict a patient’s response to a given drug. However, experts can use their experience to judge which iron chelator has better efficacy in a particular condition [26]. The effectiveness criterion is divided into three sub-criteria: reduction in liver iron concentration, reduction in cardiac iron load, and reduction in serum ferritin levels. We can determine the amount of iron overload in the patient’s body by evaluating the three sub-criteria. Therefore, it is possible to investigate the effects of a drug by analyzing the data obtained from these experiments. Knowing the worst side effects of a drug can lead to interventions that improve medical decisions, and the translation of novel therapies into clinical practice is facilitated [27].

Side effects were categorized as a criterion in the study. As the symptoms and side effects of the drugs varied among patients, this criterion was divided into three sub-criteria: severe, moderate, and mild side effects. The experts identified the mild, moderate, and severe side effects of each medication and rated them on a Likert scale from 1 to 3. The side effects were then classified using the median of the scales.

Iron chelation therapy in patients receiving regular blood transfusions may result in low patient satisfaction and compliance and may also negatively affect treatment success, patient well-being, and costs [28,29]. Therefore, in our study, patient satisfaction with the prescribed medication was a crucial criterion for selecting the most effective drug. However, the information on satisfaction was obtained from experts who have extensive clinical experience and have gained insight into patient preferences.

Cost-effectiveness is a crucial factor for prescribing treatment, as the cost of each iron chelation medication differs. It is also worth noting that since the healthcare systems of each country are different, the cost–utility analysis is one of the criteria that can be evaluated differently by the experts of each country [30,31].

### 2.3. Pairwise Comparison Matrices of FAHP

Once the hierarchical structure was complete, a pairwise comparison matrix was designed based on it. In the pairwise comparison matrix, the criteria and sub-criteria of each criterion were compared to one another with linguistic variables. Since experts mentally compare the importance of criteria, we cannot expect them to determine the exact difference between the criteria using a rigid number. Therefore, we combined fuzzy logic with the Analytic Hierarchy Process (AHP) to solve this issue. Experts use linguistic variables to compare criteria converted to triangular fuzzy numbers in FAHP [32]. We replaced the defined numbers in the pairwise comparison matrices with linguistic equivalents that ranged from strong to weak, as fuzzy logic is more akin to human logic (see Table 1).

The pair-wise comparison matrix with fuzzified triangular numbers in the FAHP method is expressed as follows:

(1) $$\tilde{A}=\left[\begin{array}{cccc} \left(1,1,1\right) & \left(a_{12},b_{12},c_{12}\right) & \ldots & \left(a_{1n},b_{1n},c_{1n}\right)\\ \left(a_{21},b_{21},c_{21}\right) & \left(1,1,1\right) & \ldots & \vdots \\ \vdots & \vdots & \ddots & \vdots \\ \left(a_{n1},b_{n1},c_{n1}\right) & \left(a_{n2},b_{n2},c_{n2}\right) & \ldots & \left(1,1,1\right) \end{array}\right]$$

Each component of the matrix is a fuzzified triangular number, with the first component (a12) being the smallest, the second (b12) being the mean, and the third (c12) being the most significant number. The sub-criteria and criteria of the alternatives were also compared to determine their weights. All questions were positively paralleled to reduce the inconsistency.

The criteria included in the hierarchical structure of the AHP are patients’ satisfaction degrees, medication efficacy, medication cost, and side effects. Sub-criteria also included the half-life of medications in plasma, cardiac iron load, liver iron concentration, serum ferritin levels, and different levels of side effects (Figure 1). Furthermore, to increase the consistency and accuracy of the pairwise comparisons, we chose to have fewer than seven criteria, and each of these criteria was also decided to have fewer than seven sub-criteria. Chang’s FAHP method was used to evaluate the completed matrices for the two groups of specialists [19].

### 2.4. Calculation of the Si for Each Row of the Pairwise Comparison Matrix

In pairwise comparison matrices, i and j are triangular fuzzy numbers representing row and column numbers. Values of $\sum_{i=1}^{n}\sum_{j=1}^{m}M_{gi}^{i}$ and ${\left[\sum_{i=1}^{n}\sum_{j=1}^{m}M_{gi}^{i}\right]}^{-1}$ are determined by applying the following formulas:

$$\tilde{S_{i}}=\sum_{j=1}^{m}\tilde{M_{g_{i}}^{j}}⊗{\left(\sum_{i=1}^{n}\sum_{j=1}^{m}\tilde{M_{g_{i}}^{j}}\right)}^{-1}$$

$$\sum_{j=1}^{m}\tilde{M_{g_{i}}^{j}}=\left(\sum_{j=1}^{m}l_{ij},\sum_{j=1}^{m}m_{ij},\sum_{j=1}^{m}u_{ij}\right)$$

$$\sum_{i=1}^{n}\sum_{j=1}^{m}M_{gi}^{i}=\left(\sum_{i,=1}^{n}l_{i}\sum_{i=1}^{n}m_{i}\sum_{i=1}^{n}u_{i}\right)$$

$${\left[\sum_{i=1}^{n}\sum_{j=1}^{m}M_{gi}^{j}\right]}^{-1}\left(\frac{1}{\sum_{i=1}^{n}u_{i}},\frac{1}{\sum_{i=1}^{n}m_{i}},\frac{1}{\sum_{i=1}^{n}l_{i}}\right)$$

### 2.5. Calculation of the Magnitude of Triangular Fuzzy Numbers

**Figure 2:**
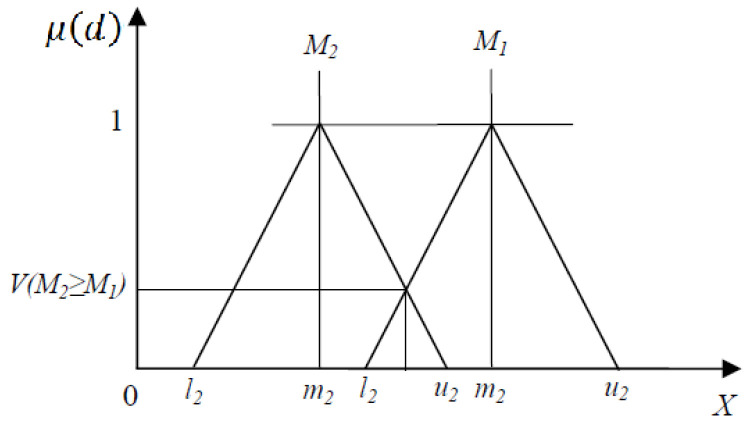
Comparison of Fuzzy Sets.

The first, second, and third components of fuzzy numbers are *l_i_*, *m_i_*, and *u_i_*. Figure 2 illustrates the comparison of fuzzy sets. *M*_1_ = (*l*_1_, *m*_1_, *u*_1_) and *M*_2_ = (*l*_2_, *m*_2_, *u*_2_) are two triangular fuzzy numbers, the magnitude of *M*_1_ with respect to *M*_2_ can be calculated as follows:

$$\mu (d)=\{\begin{array}{cc} 1 & m_{2}≧m_{1}\\ \frac{u_{1}-l_{2}}{\left(u_{1}-l_{1})-(m_{2}-l_{2}\right)} & otherwise\\ 0 & l_{1}≧u_{2} \end{array}$$

The following formula can be used to determine the magnitude of a triangular fuzzy number derived from *k* as another triangular fuzzy number:

$$V\left(M\geq M_{1},M_{2},\ldots ,M_{k}\right)=V\left[\left(M\geq M_{1}\right)\wedge \left(M\geq M_{2}\right)\wedge \ldots \left(M\geq M_{1k}\right)\right]=\min V\left(M\geq 1\right),\;I=1,2,\ldots ,k$$

### 2.6. Calculation of the Weight of the Alternatives, Criteria, and Weight Vector

To determine the weight of the criteria and alternatives, we used the formula below:

$$d^{\left(\prime \right)}\left(A_{i}\right)=\min V\left(S_{i}\geq S_{k}\right),\;k=1,2,\ldots ,n,\;k\neq i$$

Finally, the following formulas were used to calculate the normalized and non-normalized weight vectors:

$$W^{\prime }={\left(d^{\prime }\left(A_{1}\right),d^{\prime }\left(A_{2}\right),\ldots ,d^{\prime }\left(A_{n}\right)\right)}^{T},\;A_{i}\;\left(i=1,2,\ldots ,n\right)$$

$$W={\left(d\;\left(A_{1}\right),d\;\left(A_{2}\right),\ldots ,d\;\left(A_{n}\right)\right)}^{T}$$

Additionally, the experts were retrained to correct matrices if the pairwise comparison matrix’s “consistency ratio” (CR) was greater than 0.1. Thanks to the CR, we could test the study’s reliability and prevent users from making incorrect decisions. The “Consistency Index” (CI) and the “Random Consistency Index” (RI) are compared in the CR. The RI for different numbers of criteria is presented in Table 2.

CR = CI/RI

$$CI=\frac{\lambda_{max}-n}{n-1}$$

By averaging the matrix eigenvector, it is possible to determine the matrix’s highest eigenvalue (*λ_max_*). The weighted sum vector elements are divided by the corresponding priority value to determine the matrix eigenvector.

The Bland–Altman plot was conducted to compare the preferences of two experts with either over or below 10 years of experience using SPSS software version 20.

## 3. Results

This study involved a total of 20 experts, comprising 10 men and 10 women. The experts who participated in the study had an average age of 44.5 years and an average experience of 8.7 years. Using pairwise comparison matrices, the experts determined the priority and sub-criteria weights, as well as the criteria presented in Table 3. The effectiveness of the medicine was the main criterion, and its weight was 25%, 21%, and 19% higher than cost, side effects, and satisfaction, respectively. The main sub-criterion of side effects was severe side effects, which were 2.3 times more important than moderate side effects and 7.2 times more critical than mild side effects. The reduction in liver iron concentration was 1.7 and 1.9 times more important than the reduction in cardiac iron load and serum ferritin, respectively.

The priorities and the weights of the criteria and sub-criteria of each iron chelator are presented in Table 4. From the specialists’ point of view, DFP was chosen as the chelator agent with the highest priority in terms of effectiveness (1.42 and 1.07 times more than DFX and DFO, respectively). DFX was preferred to the other medications in terms of price (4.13 and 4.34 times more than DFO and DFP, respectively) and patient satisfaction (3.85 and 3.53 times more than DFO and DFP, respectively). Concerning the side effects, DFO was the preferred medication (1.31 and 1.13 times more than DFX and DFP, respectively).

In terms of reducing the iron concentration in the liver, DFO was 22.34 and 2.85 times more effective than DFX and DFP, respectively. DFP was 23.69 and 16.74 times more effective than DFX and DFO, respectively, in reducing the cardiac iron load. In addition, DFX is 4.71 and 9.41 times more effective than DFO and DFP, respectively, in reducing serum ferritin levels. DFO was considered the superior medication in terms of its moderate and severe side effects, as per the sub-criterion of side effects.

Table 5 also separately presents the opinions of the two specialist groups on the priority and weights of each criterion and sub-criterion. Cost and side effects are more important criteria for specialists with more than ten years of experience than for those with less experience.

Regarding the views of the two groups, there were some differences in the results obtained from the matrices related to the criteria and sub-criteria of each iron chelator, as shown in Table 6. Regarding effectiveness, DFO and DFP were preferred as the most effective medications by specialists with over or below ten years of experience, respectively. In the sub-criteria of “reduction in cardiac/liver iron load”, specialists with more than ten years of experience gave the most scores to DFP. Meanwhile, DFX received the highest scores in the sub-criterion of “reduction in serum ferritin”. However, the less experienced specialists gave the highest scores to DFO in the criteria.

**Figure 3:**
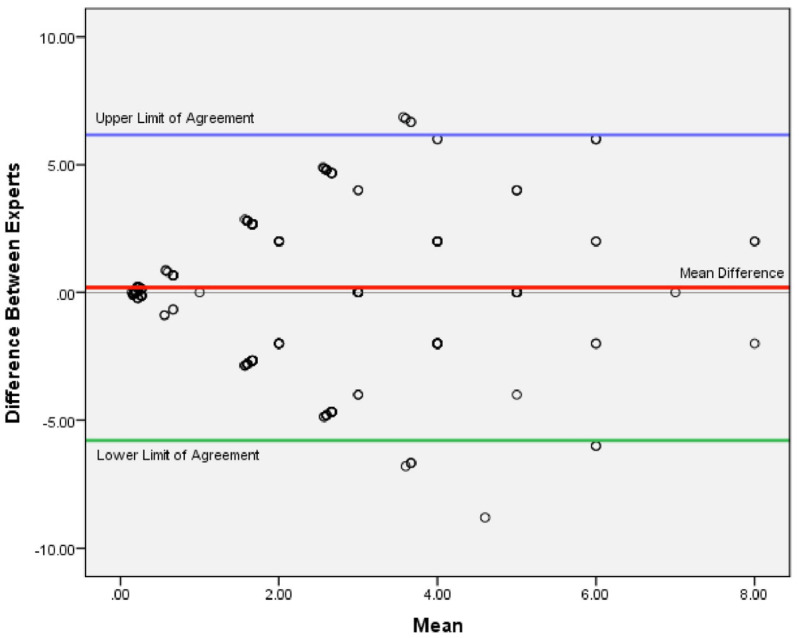
The Bland–Altman plot is used to test the agreement between two groups of experts.

Although the results from the two groups’ perspectives varied somewhat, the statistical analysis findings showed no statistically significant differences (*p* = 0.363). We used the Bland–Altman method to assess the agreement between the preferences of the two expert groups (Figure 3). To perform this test, we needed to ensure that the data distribution was normal and there were no significant differences between the groups, which was confirmed. Circles in the figure represent individual data points. Figure 3 indicates that the upper and lower limits of the mean difference do not have any proportional bias. Furthermore, the linear regression results confirm the agreement between the views of the two expert groups.

**Figure 4:**
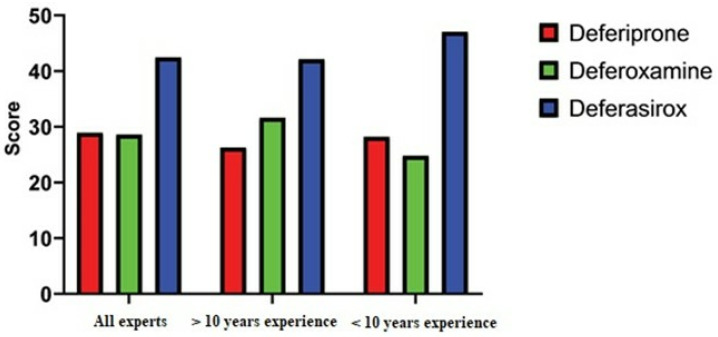
The scores and ranking of the iron chelator medications.

In conclusion, DFX was chosen as the best iron-chelating drug among all the specialists, and the other two drugs were believed to have no difference from each other (DFX was considered to be 14% superior to the other two). The scores and ranking of the iron chelator medications are shown in Figure 4 based on the expert’s judgments.

## 4. Discussion

In this study, we ranked the iron chelator medicines and listed the criteria and sub-criteria that affect the medicine preference based on the FAHP method. Although FAHP has been widely used in various fields of medicine and the health sciences, and has proven to be an efficient method, it has not yet been applied to decision making regarding iron chelation medications [15]. The specialists have three options for iron chelation medication because there is no explicit guideline for selecting the most suitable iron chelators to manage iron overload, and they are unsure which medication is more appropriate for each patient. AHP can be utilized in these situations to facilitate group decision making and help them balance the strengths and weaknesses of each alternative or medication. Furthermore, AHP provides a framework for assessing the relative weights and effects of treatment-related outcomes, offering a clear starting point for decision making.

Additionally, we investigated whether specialists’ experience affects the medications prescribed. We assumed that more experienced specialists would give higher scores to older medications. To assess this hypothesis, hematologists are divided into two groups: specialists with more than ten years of experience and those with less working experience. Results demonstrated that working experience does not affect the ranks of alternative medicines. Transfusion-dependent thalassemia patients must select an appropriate iron chelator to reduce the body’s iron burden, which can lead to a longer lifespan and improved quality of life. In this study, the specialists gave higher scores to DFX than DFP and DFO, based on the FAHP method. This means DFX is the best choice for managing chronic iron overload in thalassemia patients. Based on evidence from clinical trials, the experiences of clinicians and patients, and the financial implications of various treatment options, deferasirox was the first treatment choice for iron overload.

It has been demonstrated that DFX provides a reliable treatment for reducing the iron burden in patients with severe iron overload. It was approved by the “European Medicines Agency” (EMA) and the “US Food and Drug Administration” (FDA) in 2005. DFX at 20 or 30 mg/kg/day had a positive effect on serum ferritin levels and liver iron concentration; the tolerability profile for this medication was manageable with regular patient monitoring [33]. However, considering the complications related to inhibiting iron overload and the adverse effects of DFX treatment on renal function and other severe side effects, a long-term assessment of its efficacy and tolerability is required. Investigations have shown that a wider group of patients reported that treatment with DFX is more convenient, satisfactory, and less time-consuming compared to infusional DFO [34,35].

The measurement of cardiac iron concentration is necessary for all thalassemia patients, regardless of their liver iron concentration or serum ferritin levels [36]. In our study, the FAHP indicated that DFP has the most impact on cardiac iron overload. Some other research also confirms this result. Patients who received oral DFP had a lower iron burden in their hearts and a significantly higher left ventricular ejection fraction [37,38]. In a similar vein, Pepe et al. compared the effects of DFP, DFX, and DFO on myocardial iron overload in a large group of thalassemia patients. Patients were divided into three groups: 24 patients treated with DFX, 42 patients treated with DFP, and 89 patients treated with DFO, respectively. Patients who received DFP exhibited lower myocardial iron overload and enhanced systolic ventricular function [39]. Regarding reducing the myocardial iron burden, DFX and DFO have no superiority over each other [40].

Liver iron concentration (LIC) accurately predicts total body iron stores [41]. LIC should be measured annually in patients who receive regular blood transfusions [36]. Our results indicated that DFO is more efficient in reducing liver iron concentration than the other two medications. Similarly, Pepe et al. have reached the same conclusion [39]. Some investigations stated that DFX has different effects depending on its dosage. The groups treated with 5 to 10 mg of DFX showed no reduction in liver iron concentration. However, this was observed in the groups treated with 20 mg and 30 mg of DFX, even though the analysis of the two subgroups in this article revealed no statistically significant differences in liver iron concentration levels between the DFP and DFO treatment groups [8].

Serum ferritin measurement is a simple way to assess iron burden, and it is indispensable for evaluating the effectiveness of iron chelators. It is beneficial to analyze the changes in iron burden during the regular monitoring of patients [36]. Our results indicated that DFX is more efficient in reducing serum ferritin levels. According to a meta-analysis study, the decrease in serum ferritin levels in the DFX treatment group was greater than in the DFO treatment group. Furthermore, a combined analysis of six trials comparing DFO- and DFP-treated groups revealed no significant differences between the two groups [8].

Patients’ satisfaction with their medication is an index to improve the quality of healthcare [42]. It appears to be connected with patients’ adherence to the prescribed medications, which are required to manage iron overload toxicities [43]. Most documents revealed that DFX-treated patients are more satisfied with the DFX treatment, which is more convenient and less time-consuming than DFO treatment [44]. A randomized clinical trial was performed to compare the safety and efficacy of DFX and DFO. Ninety percent of the DFX group stated that DFX was convenient, while sixty percent of the DFO group reported that DFO was inconvenient. The main reasons for choosing DFX were as follows: being more convenient to take, causing less pain, and having fewer interferences with daily activities [31,45]. These findings proposed that DFX could be advantageous to patients who have previously failed to comply with infusional DFO [44].

When it comes to prescribing a treatment, cost-effectiveness becomes an essential factor in decision making, as there are price differences between these treatments [46]. In our investigation, the participants judged DFX as the most cost-effective medication for controlling iron overload. Cost–utility studies conducted in the UK and the US regarding iron-chelating therapy in patients with sickle cell anemia, thalassemia, and myelodysplastic syndrome recommended DFX treatment over DFO treatment, as it is more cost-effective [47].

Recent findings indicated that DFX is more cost-effective than DFO for Iranian patients. In another study in Italy, the cost-effectiveness of chelating medications was compared with that of other medications using a Markov model. Their results showed that DFP provides more advantages in terms of cost-effectiveness to the Italian healthcare system [48]. As the cost–utility analysis depends on many factors associated with each country’s healthcare system, further investigation is needed to calculate costs and QALYs for Iron chelators in the Iranian health system to approve these results in Iran.

Thalassemia patients are routinely tested for iron toxicity and the effects of their underlying conditions. Laboratory tests regularly to evaluate organ function include serum ferritin levels, echocardiography, liver enzyme levels, and serum Zn levels [49,50]. Chelator toxicity is a significant concern in iron chelation therapy, particularly in patients with depleted iron stores. Thus, the regular monitoring of chelating medication toxicity is essential for these patients [51]. In several studies, some adverse effects were indicated for each medication. The specialists who participated in this study assessed these adverse effects by determining their severity using the following classification: severe, moderate, and mild.

The already known adverse effects of DFX treatment in thalassemia patients include mild gastrointestinal symptoms (nausea, stomach pain, or diarrhea), generalized rash at high doses, and a moderate rise in creatinine levels in about a third of the people [33]. Some adverse effects, such as local skin reactions and toxicities, including hearing problems and eye disorders (optic neuropathy, retinal pigmentation), are stated in DFO-treated patients. Rare side effects have included growth retardation, renal insufficiency, pulmonary fibrosis, and anaphylactic reactions [52]. DFP is known to cause gastrointestinal symptoms, including abdominal pain, nausea, and vomiting, as well as a temporary increase in liver enzyme levels, neutropenia, arthropathy, agranulocytosis, and zinc deficiency in individuals with diabetes. The rate of these side effects is unrelated to the level of iron overload. Thrombocytopenia is a significant side effect of young thalassemia [53,54]. Generally, DFO was judged to have fewer side effects and be safer than DFX and DFP. The most effective and safest iron chelator has yet to be identified; therefore, more long-term and large-scale studies are needed to clarify this matter.

## 5. Conclusions

In conclusion, this study represents a pioneering effort in applying the “Fuzzy Analytic Hierarchy Process” (FAHP) to inform decisions regarding iron chelation medications in the context of thalassemia. In this area, FAHP has not been previously utilized. While the FAHP method has been used in decision making within the healthcare and diabetes medication settings, its application in guiding iron chelation medication decisions represents a novel endeavor.

The investigation conducted in this study explored eliciting and assessing experts’ preferences regarding diverse criteria and sub-criteria related to iron-chelating medications for thalassemia patients using the FAHP method. Our findings suggest that, through the FAHP model, deferasirox (DFX) emerged as the preferred choice for managing chronic iron overload in thalassemia patients, obtaining higher scores compared to deferiprone (DFP) and deferoxamine (DFO). While these findings provide critical insights for clinical decision making, we explicitly recommend multinational validation studies across diverse healthcare systems (e.g., public and private models, varying resource settings) and patient populations (pediatric and adult, different transfusion regimens) to confirm generalizability.

## Figures and Tables

**Table 1:** Equivalent fuzzy numbers for each linguistic variable.

Linguistic Variables	Triangular Fuzzy Numbers	Triangular Fuzzy Numbers (Reciprocals)
Equal importance	(1, 1, 1)	(1, 1, 1)
Moderate importance	(2, 3, 4)	(1/4, 1/3, 1/2)
Strong importance	(4, 5, 6)	(1/6, 1/5, 1/4)
Very strong importance	(6, 7, 8)	(1/8, 1/7, 1/6)
Extreme importance	(9, 9, 9)	(1/9, 1/9, 1/9)
Intermediate values	(1, 2, 3), (3, 4, 5), (5, 6, 7), and (7, 8, 9)	(1/3, 1/2, 1), (1/5, 1/4, 1/3), (1/7, 1/6, 1/5), and (1/9, 1/8, 1/7)

**Table 2:** RI for different numbers of criteria.

N	2	3	4	5	6	7	8	9	10
RI	0.00	0.58	0.9	1.12	1.24	1.32	1.41	1.45	1.51

N: Number of items.

**Table 3:** Criteria and sub-criteria weights.

	Criteria and Sub-Criteria	Normalized Weight	Dimension	RI	λ ^1^	CI ^2^	CR ^3^
Criteria	Effectiveness	41.43	4	0.9	4.24	0.08	0.088
	Satisfaction	22.27					
	Side effects	20.19					
	Cost	16.11					
Side effects sub-criteria	Severe side effects	63.81	3	0.58	3.06	0.03	0.052
	Moderate side effects	27.37					
	Mild side effects	8.82					
Effectiveness sub-criteria	Reduction in liver iron concentration	47.09	3	0.58	3.10	0.05	0.088
	Reduction in cardiac iron load	27.75					
	Reduction in serum ferritin	25.16					

^1^: Eigenvalue; ^2^: Consistency Index; ^3^: Consistency Ratio.

**Table 4:** The criteria and sub-criteria weights of each iron chelator.

	Deferasirox	Deferoxamine	Deferiprone	Dimension	RI	λ	CI	CR
Effectiveness	26.70	35.25	38.05	3	0.58	3.02	0.013	0.022
Reduction in liver iron concentration	19.39	41.73	38.88	3	0.58	3.02	0.012	0.021
Reduction in cardiac iron load	23.12	30.07	46.81	3	0.58	3.02	0.013	0.023
Reduction in serum ferritin	38.04	33.33	28.63	3	0.58	3.03	0.017	0.029
Satisfaction	67.94	16.44	15.62	3	0.58	3.02	0.013	0.022
Side effects	28.85	37.84	33.31	3	0.58	3.03	0.013	0.026
Mild side effects	29.85	33.29	37.86	3	0.58	3.03	0.018	0.031
Moderate side effects	28.63	40.08	31.30	3	0.58	3.02	0.014	0.025
Severe side effects	27.21	39.98	32.81	3	0.58	3.02	0.013	0.023
Cost	64.84	16.84	18.33	3	0.58	3.02	0.013	0.022

**Table 5:** The weights of each criterion and sub-criterion from the point of view of two groups of specialists.

Criteria and Sub-Criteria	D	RI	Weight for Experience < 10	λ	CI	CR	Weight for Experience > 10	λ	CI	CR
Effectiveness	4	0.9	41.62	4.19	0.063	0.070	29.72	4.21	0.071	0.079
Satisfaction			24.97				23.30			
Side effects			15.86				21.06			
Cost			17.55				26.01			
Severe side effects	3	0.58	63.32	3.05	0.025	0.043	64.52	3.08	0.041	0.070
Moderate side effects			28.44				25.58			
Mild side effects			8.24				9.90			
Reduction in liver iron load	3	0.58	52.83	3.04	0.023	0.04	60.67	3.05	0.025	0.044
Reduction in cardiac iron load			28.66				20.07			
Reduction in serum ferritin			18.51				19.26			

**Table 6:** Criteria and sub-criteria weight of each iron chelator in the view of the two groups of specialists.

	Deferasirox		Deferoxamine		Deferiprone	
	<10	>10	<10	>10	<10	>10
Cost	62	60	15	23	23	16
Effectiveness	23	35	44	26	33	38
Reduction in liver iron concentration	16	28	48	32	36	40
Reduction in cardiac iron load	21	29	43	23	36	48
Reduction in serum ferritin	31	49	41	25	27	26
Satisfaction	67	69	19	13	15	18
Side effects	32	23	38	37	30	41
Mild side effects	33	25	32	36	35	38
Moderate side effects	34	21	37	45	29	35
Severe side effects	29	23	46	29	25	48

## Data Availability

The datasets used in this study are available upon reasonable request.
